# Blood Urea Nitrogen–to–Serum Albumin Ratio Predicts Fatal Outcomes in Severe Fever with Thrombocytopenia Syndrome Patients

**DOI:** 10.4269/ajtmh.23-0811

**Published:** 2024-05-28

**Authors:** Kangli Cao, Along Ma, Ke Zhang, Yuntao Zhang, Xinjian Xiang, Cairui Xu, Binhang Han, Yuanhong Xu, Ling Tang

**Affiliations:** ^1^ Department of Clinical Laboratory, The First Affiliated Hospital of Anhui Medical University, Hefei, China;; ^2^ The First Clinical Medical School of Anhui Medical University, Hefei, China;; ^3^ Department of Plastic and Reconstructive Surgery, The Second Affiliated Hospital of Anhui Medical University, Hefei, China

## Abstract

There are no effective therapies for severe fever with thrombocytopenia syndrome (SFTS), and existing predictors of mortality are still controversial. This retrospective study aimed to identify reliable early-stage indicators for predicting fatal outcomes in 217 patients hospitalized with an SFTS diagnosis between March 2021 and November 2023; 157 of the patients survived, and 60 died. Demographics, clinical characteristics, and laboratory parameters were reassessed in both groups. The mean age of participants was 64.0 (interquartile range: 54.5–71.0) years, and 42.4% (92/217) were males. Based on a multivariate Cox regression analysis, the blood urea nitrogen–to–serum albumin ratio (BAR) (hazard ratio [HR]:4.751; 95% CI: 2.208–10.226; *P* <0.001), procalcitonin level (HR: 1.946; 95% CI: 1.080–3.507; *P* = 0.027), and central nervous system symptoms (HR: 3.257; 95% CI, 1.628–6.513; *P* = 0.001) were independent risk factors for mortality in SFTS patients. According to a receiver operating characteristic curve analysis, a BAR with an area under the curve of 0.913 (95% CI: 0.873–0.953; *P* <0.001), a sensitivity of 76.7%, and a specificity of 90.4% showed better predictive performance for fatal outcomes than other classical indicators reported. The Kaplan-Meier survival curve confirmed that an increased BAR was linked with an unfavorable prognosis in SFTS patients (*P* <0.001 by log-rank test). In conclusion, the results indicate that high BAR levels are markedly related to substandard outcomes and are a reliable and readily accessible predictor of fatal outcomes in SFTS patients.

## INTRODUCTION

Severe fever with thrombocytopenia syndrome (SFTS) is a lethal tick-borne sickness induced by the SFTS virus (SFTSV), classified as *Bunyaviridae*. Clinically, it is a broad-spectrum disease that was first identified in China in 2009 and promptly spread worldwide.^1^ Sporadic cases are reported primarily in spring and summer, mainly in mountainous and hilly rural areas. The SFTSV is believed to be a zoonotic virus^2^ that spreads by ticks to people, with this being the predominant route of transmission. In addition, the ability of SFTS to be transmitted between people through bodily fluids has been demonstrated.^3^

Patients suffering from SFTS present with multiple clinical manifestations, including fever, thrombocytopenia, gastrointestinal tract symptoms, neurological manifestations, and disseminated intravascular coagulopathy.^4^ The clinical process of SFTS can be categorized into three distinct phases depending on the progression of the illness: the first onset of fever, followed by multiple organ dysfunction, and eventually the recovery phase.^5^ This acute hemorrhagic febrile illness that results from SFTSV has a high death rate, varying from 11.2% to 30%, and is still poorly understood in terms of its pathophysiology, and there is currently no known cure.^1^^,^^6^ To forecast catastrophic consequences at an early stage of SFTS, close monitoring of some practical yet reliable indicators is essential. Some evidence suggests that a variety of markers are indicative of the outcome of SFTS patients, such as the C-reactive protein (CRP)–to–albumin ratio (CAR),^7^ the platelet-to-albumin ratio (PAR),^8^ and the aggregate index of systemic inflammation (AISI), which represents immunological and inflammatory status as well as the associated infectious disease risk and mortality.^9^ However, the diagnostic efficiency of these markers varies according to different datasets. On the other hand, several scoring models comprising specific hard-to-obtain clinical parameters have been proposed,^10^^,^^11^ although they are too complex for wide clinical application. Thus, there is an urgent need to explore novel and more effective predictors of SFTS mortality so that early management strategies and timely interventions can improve outcomes in high-risk patients.

It has been reported that the liver and kidneys are the primary organs targeted by SFTSV infection,^12^ as evidenced by alterations in relevant laboratory parameters. Blood urea nitrogen (BUN) and serum albumin (ALB) are indicators of liver and kidney function. The predictive usefulness of the BUN-to-ALB ratio (BAR) for some infectious disorders has been highlighted by research,^13^^,^^14^ and the mortality prediction of the BAR is substantially higher than that of BUN and ALB.^15^^,^^16^ However, in the available literature, no SFTS studies have used the BAR to predict SFTS mortality. Therefore, the purpose of the current research was to verify whether the BAR could be used as an early diagnostic indicator in SFTS patients and whether it is superior to other existing predictors.

## MATERIALS AND METHODS

### Study strategy and subjects.

The current retrospective research involved SFTS patients hospitalized at the top two large tertiary hospitals in Anhui Province (the First and Second Affiliated Hospitals of Anhui Medical University) during March 2021 and November 2023. For inclusion, all of the following criteria had to be met: 1) acute fever with thrombocytopenia; 2) laboratory-confirmed SFTSV infection using a real-time fluorescence polymerase chain reaction nucleic acid test; and 3) age older than 18 years. Patients who met any of the following criteria were excluded: 1) a history of autoimmune diseases; 2) a chronic hematological disease; 3) a chronic liver disease; 4) end-stage renal failure or hemodialysis; 5) persistent immunosuppression from an infection with HIV, radiation or chemotherapy for cancer, or other immunosuppressive therapy; 6) concomitant infection with the Epstein-Barr virus, rickettsial infection, or other acute infection within the past month; and 7) insufficient clinical and laboratory information.

Subjects were placed into deceased and survival groups on the basis of their survival outcomes. A patient who sadly died during the period of illness was defined as deceased, whereas survivors were those whose temperature progressively recovered to norms and manifestations abated to the point where they could be discharged. All patients were followed up for 28 days after discharge. This research complied with the Declaration of Helsinki and was affirmed by the Local Research Ethics Committee of the First Affiliated Hospital of Anhui Medical University.

### Information collection.

Data from the participants’ digital medical histories taken on the day of hospitalization were retrieved employing the Hospital Information Management System program; this included patient demographics (sex, age, comorbidities, outcome), clinical manifestations, medical history, and laboratory data (including routine blood, biochemical, and coagulation markers). Blood specimens were taken within 24 hours of admission. The BUN value (mmol/L) was converted to BUN (mg/dL) by multiplying it by 2.86, whereas the ALB level (g/L) was converted to ALB (g/dL) by dividing it by 10. The BUN levels (mg/dL) were divided by the ALB levels (g/dL) to get the BAR (mg/g), and the CAR (mg/g) equaled the ratio of C‐reactive protein (CRP; mg/dL) to ALB (g/dL). The PAR equaled platelets (PLTs; 10^9^/L)-to-ALB (g/L) ratio, and the AISI equaled (neutrophils × monocytes [MONOs] × PLTs)/lymphocytes (LYMs; 10^9^/L). All information was entered by trained learning personnel. Patients who ceased therapy or were released owing to unfavorable clinical advancement or financial concerns were contacted by phone within 28 days to assess their outcomes (surviving or deceased).

### Definition.

Skin alterations were defined as the presence of any of the following: rash, skin nodules, and skin color changes. Oral gingival bleeding, petechia, ecchymosis, melena, and hemafecia were included in the definition of hemorrhage. Central nervous system (CNS) symptoms included muscle tremors, confusion, coma, convulsion, and neurological signs. Gastrointestinal signs included the following symptoms: vomiting, diarrhea, nausea, loss of appetite, abdominal distension, and abdominal pain.

## STATISTICAL ANALYSES

Data were depicted as mean *(*$\bar{x}$) ±SD or median of the interquartile range (IQR), and intergroup comparisons were analyzed by independent sample *t*-test or Mann–Whitney *U* test, respectively. Categorical variables were displayed as percentages (*n*, %) and assessed via χ^2^ or Fisher’s exact tests. To avoid the reduction of efficiency and possible bias caused by the direct exclusion of missing data, multiple imputations were conducted to estimate missing values. Univariate and multivariable Cox regression analyses with an entry procedure were applied to elucidate distinct risk factors of SFTS-related deaths. Variables with *P <*0.01 in the univariate Cox regression were subsequently included in the multivariate Cox regression to identify independent predictors of poor outcomes in SFTS patients. Hazard ratios and 95% CIs were computed. With the help of receiver operating characteristic (ROC) analysis, the predictive power of the BAR, BUN, ALB, CAR, PAR, and AISI for mortality was determined. The DeLong test evaluated the validity of models in MedCalc v. 19.2.0. The maximum Youden’s index, defined as (sensitivity + specificity − 1), was used to calculate the optimum cutoff levels with the highest accuracy. Kaplan-Meier survival analysis was performed to evaluate accumulated survival in the groups with lower and higher BAR cutoff values, and the log-rank test was conducted to confirm the validity of the discrepancy.

The SPSS 26.0 statistical software (IBM Corp., Armonk, NY) was used for statistical analysis. The graphs were produced by GraphPad Prism 9.5.1 (La Jolla, CA). The statistical significance level was established to be two-sided *P <*0.05.

## RESULTS

### Demographics and clinical features of SFTS patients.

A total of 260 patients with SFTS admitted to the hospitals between March 2021 and November 2023 were screened using the inclusion and exclusion criteria. In all, 217 individuals who matched the qualification criteria for laboratory-confirmed SFTS were included in the cohort. The flowchart of the research study is given in Supplemental Figure 1.

Of the 217 participants, 60 died of SFTS-associated manifestations, giving a mean mortality percentage of 27.6%. Patient age ranged from 30 to 86 years, and the median age at admission was 64.0 (IQR: 54.5–71.0) years. One hundred twenty-five participants (57.6%) were female. A total of 54 patients were confirmed as having a previous record of tick bites prior to the commencement of SFTS, and 10 (4.6%) became infected as a result of intimate contact with SFTS patients. Hypertension was the most common underlying condition, observed in 23.5% of patients with SFTS. Compared with the survival group, patients who died were older (*P <*0.001) and had hospital stays of shorter duration (*P <*0.001) (Table 1).

**Table 1:** Clinical characteristics and laboratory parameters of SFTS patients

Variables	Total (*N =* 217)	Survival (*n =* 157)	Deceased (*n =* 60)	*P*-Value
General Characteristics				
Sex, *n* (%)				
Male	92 (42.4)	63 (40.1)	29 (48.3)	0.274
Female	125 (57.6)	94 (59.9)	31 (51.7)	–
Age (years)	64.0 (54.5–71.0)	63.0(53.0–69.0)	69.0 (63.3–75.0)	<0.001
Duration of Hospital Stay (days)	13 (9.0–16.5)	14 (11.0–18.0)	7 (6.0–12.0)	<0.001
Tick Bite History, *n* (%)	54 (24.9)	39 (24.8)	15 (25.0)	0.981
Contact with SFTS Patients, *n* (%)	10 (4.6)	9 (5.7)	1 (1.7)	0.36
Underlying Diseases, *n* (%)				
Type 2 Diabetes	30 (13.8)	22 (14.0)	8 (13.3)	0.897
Hypertension	51 (23.5)	32 (20.4)	19 (31.7)	0.08
Malignant Tumor (history)	3 (1.4)	3 (1.9)	0	0.563
Cerebral Infarction	22 (10.1)	14 (8.9)	8 (13.3)	0.335
Clinical Manifestations, *n* (%)				
Headache	31 (14.3)	20 (12.7)	11 (18.3)	0.292
Skin Changes	27 (12.4)	22 (14.0)	5 (8.3)	0.257
Fatigue	170 (78.3)	123 (78.3)	47 (78.3)	0.999
Arthralgia	8 (3.7)	5 (3.2)	3 (5.0)	0.817
Myalgia	78 (35.9)	54 (34.4)	24 (40.0)	0.442
Chest Distress	24 (11.1)	13 (8.3)	11 (18.3)	0.035
Palpitation	19 (8.8)	11 (7.0)	8 (13.3)	0.14
Gastrointestinal Symptoms	185 (85.3)	131 (83.4)	54 (90.0)	0.223
Hemorrhage	51 (23.5)	30 (19.1)	21 (35.0)	0.014
CNS Symptoms	67 (30.9)	21 (13.4)	46 (76.7)	<0.001
Outcomes, *n* (%)				
Admitted to ICU	7 (3.2)	0	7 (11.7)	<0.001
7-Day Mortality	32 (14.7)	0	32 (53.3)	<0.001
Laboratory Parameters				
WBC (3.5–9.5 ×10^9^/L)	2.63 (1.65–4.56)	2.72 (1.68–4.87)	2.43 (1.54–3.58)	0.121
NEU (1.8–6.3 ×10^9^/L)	1.67 (0.95–3.02)	1.67 (0.95–3.24)	1.67 (0.86–2.55)	0.449
LYM (1.1–3.2 ×10^9^/L)	0.70 (0.45–1.08)	0.75 (0.48–1.17)	0.56 (0.41–0.81)	0.014
MONO (0.1–0.6 ×10^9^/L)	0.13 (0.07–0.29)	0.15 (0.08–0.33)	0.09 (0.07–0.16)	0.003
PLTs (125–350 ×10^9^/L)	49.00 (36.00–71.50)	56.00 (40.00–81.00)	40.00 (29.25–52.50)	<0.001
ALB (4.0–5.5 g/dL)*	3.52 ± 0.59	3.63 ± 0.62	3.26 ± 0.40	<0.001
ALP (50–135 U/L)	70.00 (56.00–90.50)	69.00 (55.00–83.50)	77.00 (58.00–112.25)	0.044
ALT (7–40 U/L)	73.00 (47.00–113.50)	65.00 (40.50–93.00)	118.50 (73.00–184.25)	<0.001
GGT (7–45 U/L)	35.00 (21.50–69.50)	34.00 (19.50–62.50)	36.00 (24.25–96.00)	0.069
TB (0–23 µmol/L)	10.40 (8.15–14.30)	10.50 (8.15–14.30)	10.30 (8.10–14.80)	0.907
BUN (9.15–20.31 mg/dL)	18.67 (14.24–25.89)	15.73 (13.15–20.88)	28.18 (22.13–36.39)	<0.001
eGFR (>90 mL/(min·1.73 m^2^)	93.00 (69.50–103.50)	96.00 (86.50–107.00)	63.00 (40.00–87.50)	<0.001
CK (30–135 U/L)	388.50 (139.50–975.75)	290.50 (124.75–722.75)	775.00 (420.50–2796.25)	<0.001
HCO_3_^−^ (22–34 mmol/L)*	23.93 ± 4.05	24.86 ± 3.31	21.50 ± 4.76	<0.001
LPS (23–300 U/L)	575.00 (304.00–1008.00)	523.00 (253.50–890.00)	860.00 (481.50–1402.50)	<0.001
AMY (30–110 U/L)	106.00 (71.00–184.00)	93.00 (63.00–142.00)	166.50 (96.75–262.25)	<0.001
CRP (0–0.3 mg/dL)	2.80 (0.83–10.28)	2.20 (0.67–7.97)	5.92 (2.10–15.83)	0.002
PCT (<0.5 ng/mL)	0.18 (0.07–0.62)	0.13 (0.06–0.26)	0.73 (0.25–1.59)	<0.001
BAR (mg/g)	5.37 (3.91–7.35)	4.65 (3.61–5.70)	8.87 (6.89–11.32)	<0.001
PT (11–16 seconds)	13.30 (12.70–14.10)	13.20 (12.70–13.80)	13.50 (12.95–14.50)	0.005
FDP (0–5 µg/mL)	8.95 (4.01–18.09)	6.73 (3.39–13.21)	19.7 (10.89–44.94)	<0.001

ALB = serum albumin; ALP = alkaline phosphatase; ALT = alanine transaminase; AMY = amylase; BAR = BUN-to-ALB ratio; BUN = blood urea nitrogen; CK = creatine phosphokinase; CNS = central nervous system; CRP = C-reactive protein; eGFR = estimated glomerular filtration rate; FDP = fibrinogen degradation product; GGT = gamma glutamine transferase; ICU = intensive care unit; IQR = interquartile range; LPS = lipase; LYM = lymphocyte; MONO = monocyte; NEU = neutrophil; PCT = procalcitonin; PLT = platelet; PT = prothrombin time; SFTS = severe fever with thrombocytopenia syndrome; TB = total bilirubin; WBC = white blood cell. The group was divided according to survival or deceased. *P*-values describe the comparison between survival and deceased groups. ALB (g/dL) = ALB (g/L) × 0.1; BUN (mg/dL) = BUN (mmol/L) × 2.86; BAR (mg/g) = BUN (mg/dL)/ALB (g/dL). Continuous variable data are presented as mean (SD) and median (IQR).

* The *t*-test was used to compare groups.

Gastrointestinal symptoms were the predominant clinical manifestation, exhibited by 185 patients (85.3%) in the initial stage of the illness, followed by fatigue (170/217, 78.3%). The incidence rates of chest distress (*P =* 0.035), hemorrhage (*P =* 0.014), and CNS symptoms (*P <*0.001) were more prevalent in the deceased group than in the survival group. Furthermore, intensive care units (ICU) admissions and 7-day mortality were substantially enhanced in the deceased group compared with the survivors (*P <*0.001) (Table 1).

### Comparative analysis of laboratory traits in the survival and deceased groups.

Laboratory indexes, including complete blood counts, biochemical analysis, coagulation tests, and infection-related biomarkers, are listed in Supplemental Table 1. When comparing laboratory measurements of SFTS patients in the survival and deceased groups (Table 1), we discovered that the deceased patients had considerably lower ALB, HCO_3_^−^, and estimated glomerular filtration rate (eGFR) levels and decreased counts of LYMs, MONOs, and PLTs. Meanwhile, alkaline phosphatase (ALP), alanine transaminase (ALT), BUN, creatine phosphokinase (CK), lipase (LPS), amylase (AMY), CRP, procalcitonin (PCT), BAR, prothrombin time (PT), and fibrinogen degradation product (FDP) levels were visibly higher in the fatal cases. In conclusion, advanced age, thrombocytopenia, and the previously mentioned laboratory indicators appeared to be associated with mortality due to SFTS.

### The BAR was initially established as a distinct prognostic indicator in SFTS individuals.

**Figure 1.**
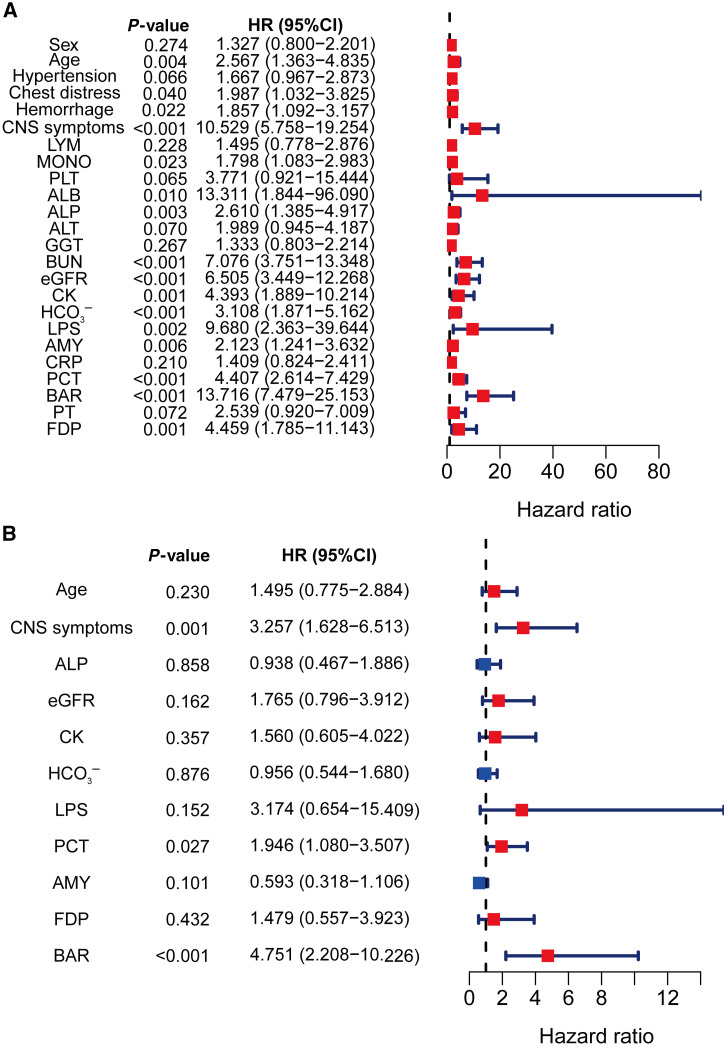
Forest plots of hazard ratios (HRs) by (**A**) univariate and (**B**) multivariable Cox regression analyses. Red indicates HR >1 and blue indicates HR <1 with 95% confidence interval (CI) as error bars. [AA] ALB = serum albumin; ALP = alkaline phosphatase; ALT = alanine transaminase; AMY = amylase; BAR = BUN-to-ALB ratio; BUN = blood urea nitrogen; CK = creatine phosphokinase; CNS = central nervous system; CRP = C-reactive protein; eGFR = estimated glomerular filtration rate; FDP = fibrinogen degradation product; GGT = gamma glutamine transferase; LPS = lipase; LYM = lymphocyte; MONO = monocyte; PCT = procalcitonin; PLT = platelet; PT = prothrombin time.

The assignment of variables for the Cox regression analysis of laboratory measures is displayed in Supplemental Table 2. A univariate Cox regression model that considered only clinically associated factors and variables with a *P*-value <0.10 was performed (Figure 1A). The results indicated that advanced age, chest distress, hemorrhage, CNS symptoms, decreased MONO counts, ALB, HCO_3_^−^, and eGFR, together with elevated levels of BAR, ALP, BUN, LPS, CK, AMY, PCT, and FDPs, were significant predictors of SFTS prognosis (*P <*0.05). After confounding factors were excluded, factors with statistically significant *P*-values <0.01 were subsequently incorporated into the multivariate Cox regression investigation (Figure 1B). Higher levels of BAR (HR: 4.751; 95% CI: 2.208–10.226; *P <*0.001) and PCT (HR: 1.946; 95% CI: 1.080–3.507; *P =* 0.027) and more frequent CNS symptoms (HR: 3.257; 95% CI: 1.628–6.513; *P =* 0.001) were identified as independent risk indicators associated with a fatal outcome in SFTS patients.

### Diagnostic performance of the BAR for mortality risk in SFTS patients.

**Figure 2.**
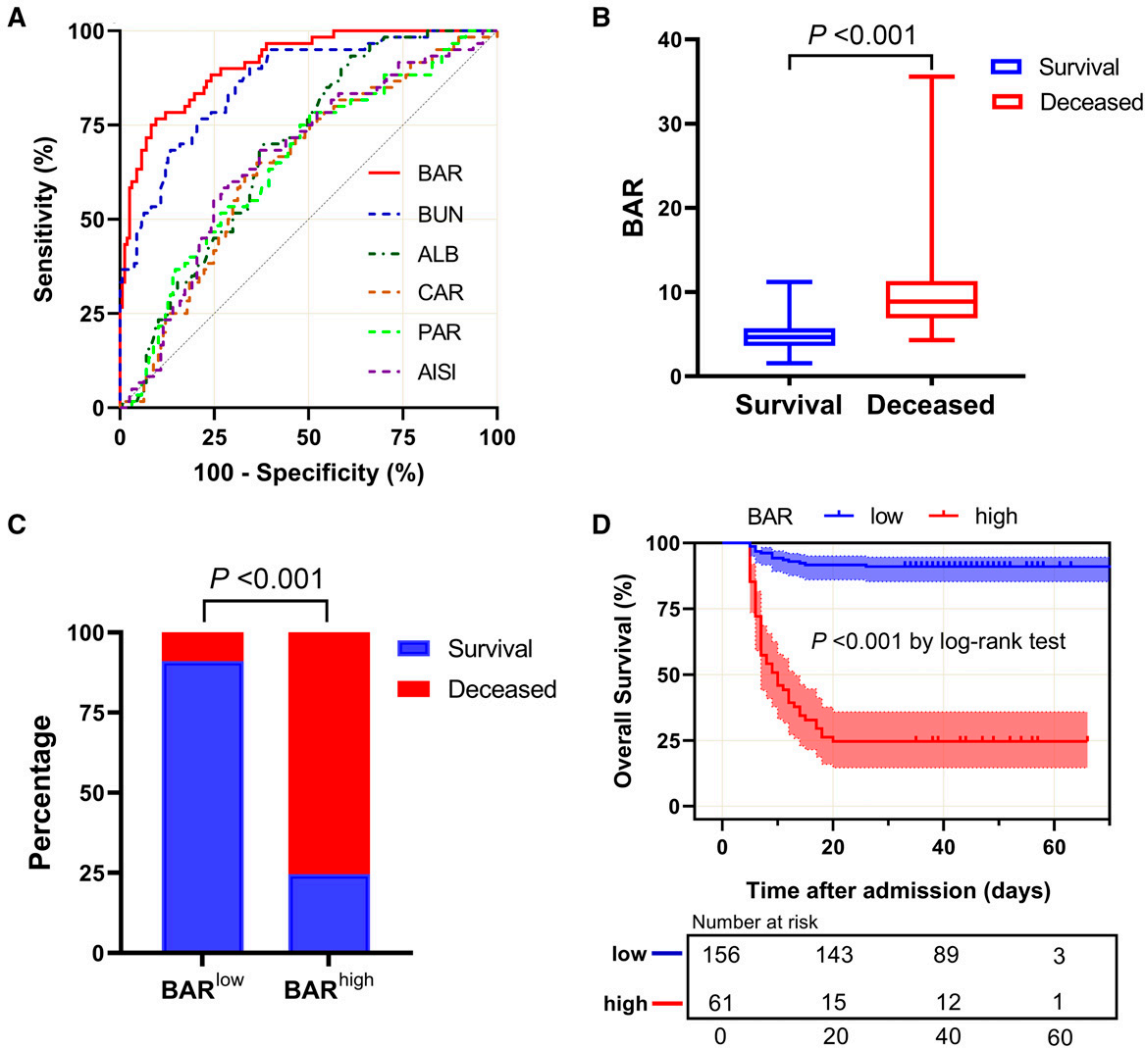
The predictive value of the BAR in SFTS patients with poor prognosis. (**A**) Receiver operating characteristic curves for evaluating the predictive value of the BAR, BUN, ALB, CAR, PAR, and AISI for poor prognosis of SFTS patients at admission. The area under the curve of the BAR is a red line, the CAR is an orange line, the PAR is a green line, the AISI is a purple line, BUN is a blue line, and ALB is a bottle green line. (**B**) Comparison of patient outcomes at various BAR levels (BAR^low^ and BAR^high^). Median as error bars. (**C**) Comparison of BAR levels between the surviving and deceased groups. (**D**) Kaplan-Meier survival curves for overall survival in SFTS patients. Patients were grouped by the cutoff value of the BAR. AISI = (neutrophils × monocytes × platelets)–to-lymphocytes ratio; ALB = serum albumin; BAR = BUN-to-ALB ratio; BUN = blood urea nitrogen; CAR = C‐reactive protein–to-ALB ratio; PAR = platelet-to-ALB ratio; SFTS = severe fever with thrombocytopenia syndrome.

To determine the diagnostic performance of the BAR on fatal outcomes in SFTS patients, the predictive efficiency of the BAR was compared with that of BUN, ALB, and several other previously reported classical indicators such as CAR, PAR, and AISI, and their optimal cutoff values were computed via ROC curve analysis. As shown in Figure 2A, the areas under the curve (AUC) for survival were 0.913 (95% CI: 0.873–0.953; *P <*0.001) for BAR; 0.862 (95% CI: 0.809–0.916; *P <*0.001) for BUN; 0.688 (95% CI: 0.617–0.759; *P <*0.001) for ALB; 0.643 (95% CI: 0.563–0.722; *P =* 0.001) for CAR; 0.652 (95% CI: 0.572–0.732; *P <*0.001) for PAR; and 0.661 (95% CI: 0.581–0.740; *P <*0.001) for AISI. In addition, the AUC of the BAR exhibited a greater magnitude than that of BUN (*Z* = 3.815, *P <*0.001), ALB (*Z* = 5.497, *P <*0.001), CAR (*Z* = 6.213, *P <*0.001), PAR (*Z* = 6.125, *P <*0.001), and AISI (*Z* = 5.500, *P <*0.001) (Supplemental Table 3). The optimum BAR cutoff value, determined by the maximum Youden’s index, was 6.712 mg/g, with a sensitivity of 76.7% and a specificity of 90.4%. It was revealed that the BAR better predicted unfavorable prognosis at admission, with a more reliable predictive performance, than the other five metrics did (Table 2).

**Table 2:** Predictive value of prognostic indicators in SFTS fatal outcomes

Parameters	AUC	Cutoff Value	Sensitivity	Specificity	95% CI	*P*-Value
BAR	0.913	6.712	0.767	0.904	0.873–0.953	<0.001
BUN	0.862	18.745	0.900	0.656	0.809–0.916	<0.001
ALB	0.688	3.755	0.933	0.389	0.617–0.759	<0.001
CAR	0.643	1.290	0.650	0.637	0.563–0.722	0.001
PAR	0.652	16.447	0.783	0.497	0.572–0.732	<0.001
AISI	0.661	14.283	0.683	0.631	0.581–0.740	<0.001

AISI = (neutrophils × monocytes × platelets)/lymphocytes ratio; ALB = serum albumin; AUC = area under the receiver operating characteristic curve; BAR = BUN-to-ALB ratio; BUN = blood urea nitrogen; CAR = C-reactive protein–to-ALB ratio; PAR = platelets-to-ALB ratio; SFTS = severe fever with thrombocytopenia syndrome. The cutoff values were selected by maximum Youden’s index (sensitivity + specificity − 1).

### The BAR was linked to fatal outcomes in SFTS patients.

It was revealed that the BAR level was notably higher in deceased patients than in those who survived (*P <*0.001) (Figure 2C). To further elucidate the predictive significance of the BAR for a 28-day prognosis, we grouped patients according to the optimum cutoff value (6.712) of the BAR: BAR^low^ and BAR^high^. As shown in Figure 2B, mortality rates were lower in SFTS participants with low BAR values than in those with high BAR values. In addition, according to the Kaplan-Meier survival curves, SFTS patients with high BAR levels had remarkably shorter survival time than those with low levels (*P <*0.001 by log-rank test) (Figure 2D).

### Pairwise correlation between the BAR and laboratory parameters.

**Figure 3.**
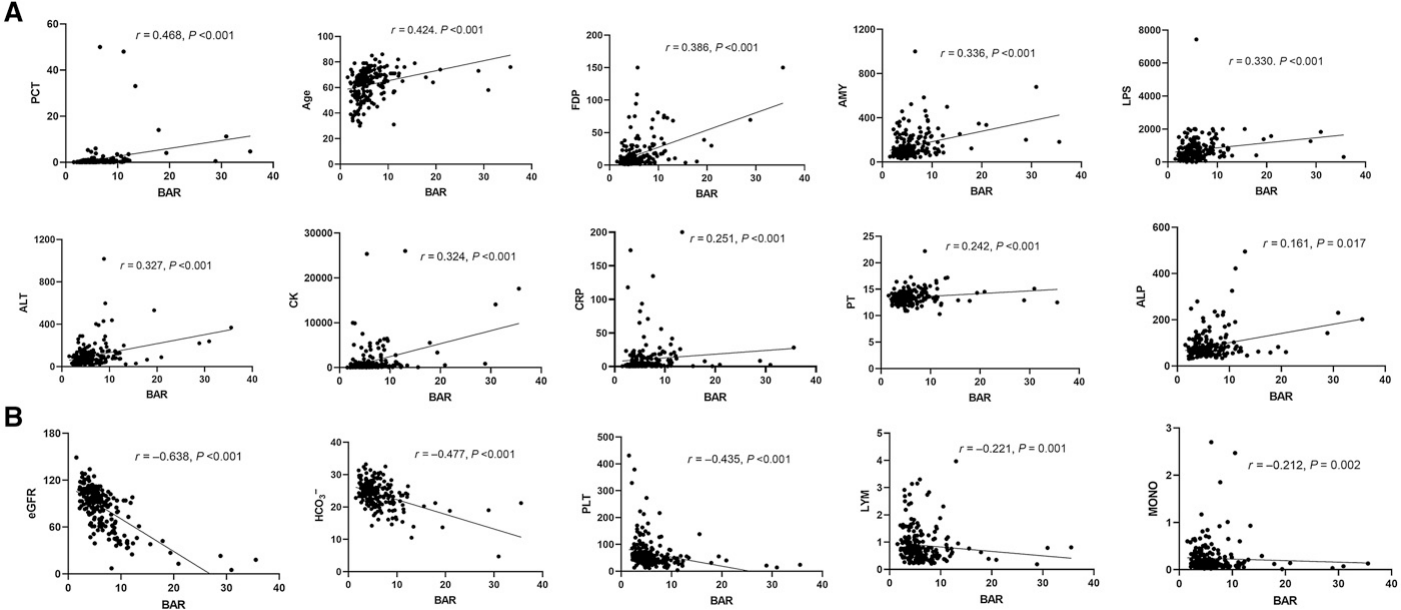
Pairwise correlation between the BAR level and clinical parameters. (**A**) Parameters with positive correlation with the BAR. (**B**) Parameters with negative correlations with the BAR. ALP = alkaline phosphatase; ALT = alanine transaminase; AMY = amylase; BAR = blood urea nitrogen–to–serum albumin ratio; CK = creatine phosphokinase; CRP = C-reactive protein; eGFR = estimated glomerular filtration rate; FDP = fibrinogen degradation product; LPS = lipase; LYM = lymphocyte; MONO = monocyte; PCT = procalcitonin; PLT = platelet; PT = prothrombin time; SFTS = severe fever with thrombocytopenia syndrome.

The aforementioned laboratory parameters differed significantly between the deceased and survival groups. Therefore, the correlation between BAR levels and these metrics was assessed. A moderate correlation was found between the BAR and PCT (*r* = 0.468, *P <*0.001), age (*r* = 0.424, *P <*0.001), FDPs (*r* = 0.386, *P <*0.001), AMY (*r* = 0. 336, *P <*0.001), LPS (*r* = 0.330, *P <*0.001), ALT (*r* = 0.327, *P <*0.001), and CK (*r* = 0.324, *P <*0.001), with a weak correlation between CRP (*r* = 0.251, *P <*0.001), PT (*r* = 0.242, *P <*0.001), and ALP (*r* = 0.161, *P =* 0.017) (Figure 3A). However, it was inversely correlated with eGFR (*r* = −0.638, *P <*0.001), HCO_3_^−^ (*r* = −0.477, *P <*0.001), PLTs (*r* = −0.435, *P <*0.001), LYMs (*r* = −0.221, *P =* 0.001), and MONOs (*r* = −0.212, *P =* 0.002) (Figure 3B). The findings indicate that the BAR level was consistent with the levels of clinical function measures.

## DISCUSSION

Severe fever with thrombocytopenia syndrome is associated with a significant mortality rate but lacks any particular therapy or vaccine. Thus, having a predictor that identifies the preliminary indications of fatal SFTS cases is of tremendous importance. In the present study, patients in the deceased group showed advanced age, specific clinical symptoms, and altered levels of laboratory indicators. Univariate and multivariable Cox regression and ROC analyses indicate that the BAR, PCT, and CNS symptoms were independent predictors of SFTS outcomes.

Central nervous system manifestations were observed in 76.7% of deceased individuals in this investigation and were a predictor of mortality in SFTS patients. The SFTSV can infect the CNS.^17^ Gai et al.^18^ reported that cases were associated with nervous system symptoms that were attributed to the seriousness of the condition, in line with the result obtained from our study. Moreover, kidney and hepatic impairments may also be related to SFTS encephalopathy.^19^ Procalcitonin, an inflammatory protein that is elevated in bacterial infections, is generally not increased in viral diseases.^20^ However, several studies have reported significantly higher PCT levels in SFTS patients than in COVID-19 patients and healthy controls.^21^ In the study conducted by Chen et al.,^22^ PCT levels were positively correlated with SFTSV load and were an inflammatory marker predicting early mortality in SFTS patients. Similarly, our study showed that PCT was an independent prognostic factor of death in SFTS patients. Laboratory tests performed within 24 hours of admission, including blood routine tests (e.g., MONOs and PLTs), coagulation function indicators (PT and FDPs), kidney function indicators (BUN and eGFR), and liver function indicators (e.g., ALT, TB, and gamma glutamine transferase), revealed significant differences between survivors and nonsurvivors. Thrombocytopenia has been reported in virtually all SFTS patients, presumably owing to the adhesion of the SFTSV to platelets and the promotion of their phagocytosis by macrophages.^23^ On the other hand, there is evidence that thrombocytopenia is associated with increased inflammatory response, endothelial damage, and coagulopathy.^24^ This finding is consistent with the observation of higher levels of PT and FDPs as well as the inflammation-related markers CRP and PCT in deceased SFTS patients in the present study. However, conflicting opinions have stated that the coagulation factors did not differ significantly between patients who survived and those who died.^18^^,^^25^ This may be due to the fact that some patients were not hospitalized in time and laboratory parameters may have changed rapidly as the disease progressed, so comparisons at different stages may lead to different conclusions.

Blood urea nitrogen is an essential index reflecting complicated interactions between patients’ nutrition, protein metabolism, and renal status.^26^ The literature links elevated BUN levels to fatalities in various diseases, such as pulmonary diseases, pancreatitis, sepsis, and COVID-19.^27^^,^^28^ Here, elevated BUN levels confirmed acute kidney injury or protein metabolism impairment in SFTSV-infected patients. Albumin, a major component of plasma proteins produced by the liver, is considered a biomarker of malnutrition and inflammation.^29^ The existing literature shows that ALB has been used as a prognostic factor of infection,^30^ and it was correlated with a risk of death in patients with SFTS in a meta-analysis.^31^ The association between decreased ALB levels and fatal outcomes from SFTS may have several explanations: Hepatic injury induced by SFTSV resulted in reduced albumin production, and a renal injury resulted in increased albumin filtration or decreased reabsorption. Interestingly, increased BUN and decreased ALB levels were observed in neonates with hereditary thrombotic thrombocytopenic purpura,^32^ severe cases of Crimean-Congo hemorrhagic fever,^33^ and fatal cases of dengue hemorrhagic fever.^34^ However, BUN and ALB were not independent hazards for SFTS patients in this study, suggesting that their efficacy alone for early identification of adverse outcomes is less than ideal. To amplify the inverse relationship between levels of BUN and ALB after SFTSV infection, the BAR, a simple index, was applied.

Per our understanding, this investigation represents an inaugural attempt to evaluate the predictive efficacy of the BAR among patients with SFTS. The results indicate that the mortality rate in patients with a high BAR (>6.712) was more than eight times greater than the rate in those with low BAR (<6.712). Because patients with chronic kidney or liver disease were excluded, this association may have been due to SFTSV-associated inflammation and direct effects of SFTSV on the liver and kidneys. The ROC examination for foreseeing SFTS mortality demonstrated that the AUC value (AUC = 0.913) and odds ratio derived from the BAR were significantly higher than those derived from BUN (AUC = 0.862) and ALB (AUC = 0.688) levels. Moreover, the CAR and PAR, which effectively recognized patients at an elevated risk of SFTS,^8^^,^^35^ and the AISI, which has been linked to the risk and mortality of infectious illnesses,^9^ were also evaluated. The results indicate that the BAR outperformed the CAR, AISI, BUN, and ALB in predicting prognosis in SFTS patients, in accordance with previous studies.^28^

The BAR is considered a composite index that reflects nutritional status, dehydration, and hepatic and renal reserve capacity.^36^ The BAR was originally developed for prognostic assessment in patients with community-acquired pneumonia.^37^ The prognostic value of the BAR as a predictive tool has been reported in some infectious diseases, such as hospital-acquired pneumonia,^13^ non-HIV *Pneumocystis* pneumonia,^36^ *Escherichia coli* blood infections,^38^ and COVID-19.^28^ Remarkably, Karasahin and Sarikaya^39^ reported that the BAR was an independent risk factor for predicting mortality from Crimean-Congo hemorrhagic fever, with high sensitivity and specificity at a cutoff of 5 mg/g. The results of this study indicate that the BAR is an independent prognostic factor in predicting mortality in SFTS patients, with a cutoff value of 6.712 mg/g and a significant positive correlation between BAR levels and age. Based on the available studies, the different cutoff values for the BAR in the two cohorts could be due to differences in age, geographic region, and/or disease stage of the enrolled patients.^40^ In addition, a significant relationship between the BAR and different clinical parameters for SFTS was also found, and the BAR inversely correlated with eGFR, HCO_3_^−^, PLT, LYM, and MONO levels. In addition, positive relationships with inflammation-related biomarkers and levels of markers commonly used to indicate impaired liver, kidney, cardiac, and pancreatic functions were also assessed, which were in line with literature confirming that SFTS in its early stages is a complex multisystem disease with acute inflammation and multiple organ dysfunction.^41^

Blood urea nitrogen and ALB are standard research facility metrics that are promptly accessible, cheap, and reproducible at admission. Considering their expansion to economically underdeveloped regions, they could rapidly identify patients at high death risk at an early stage, enabling early intervention and dynamic clinical outcome assessment of SFTS patients. Therefore, the BAR ought to be evaluated prospectively as a potential eligibility parameter for the tiered control of risk factors in SFTS individuals, as well as for adjunctive therapy trials.

This research has a few limitations. First, because of the lack of follow-up laboratory data, the BAR was detected only on hospital admission. Second, we did not validate our findings with a fresh cohort and had an insufficient sample size. Therefore, future research should involve multicenter studies with bigger cohort sizes and subsequent follow-ups and should assess whether the BAR can be used to predict infection with other viruses, particularly tick-borne viruses.

## CONCLUSION

This study compared and identified BAR, PCT, and CNS symptoms as stand-alone risk factors for mortality in SFTS patients. A significant correlation was observed between elevated BAR levels and increased mortality. Our findings demonstrate the predictive value of BAR for poor outcomes in SFTS patients and suggest that it could serve as a reliable and valid predictor of fatal outcomes in these patients.

## Supplemental Materials

10.4269/ajtmh.23-0811 Supplemental Materials
