# Supplementary material for: Blood Urea Nitrogen–to–Serum Albumin Ratio Predicts Fatal Outcomes in Severe Fever with Thrombocytopenia Syndrome Patients
Source: Am J Trop Med Hyg. 2024 May 28;111(1):113–20. doi: 10.4269/ajtmh.23-0811 (PMC11229660; doi:10.4269/ajtmh.23-0811)
Supplement: Supplemental Materials [file tpmd230811.SD1.pdf]

Supplementary Materials

Figure S1

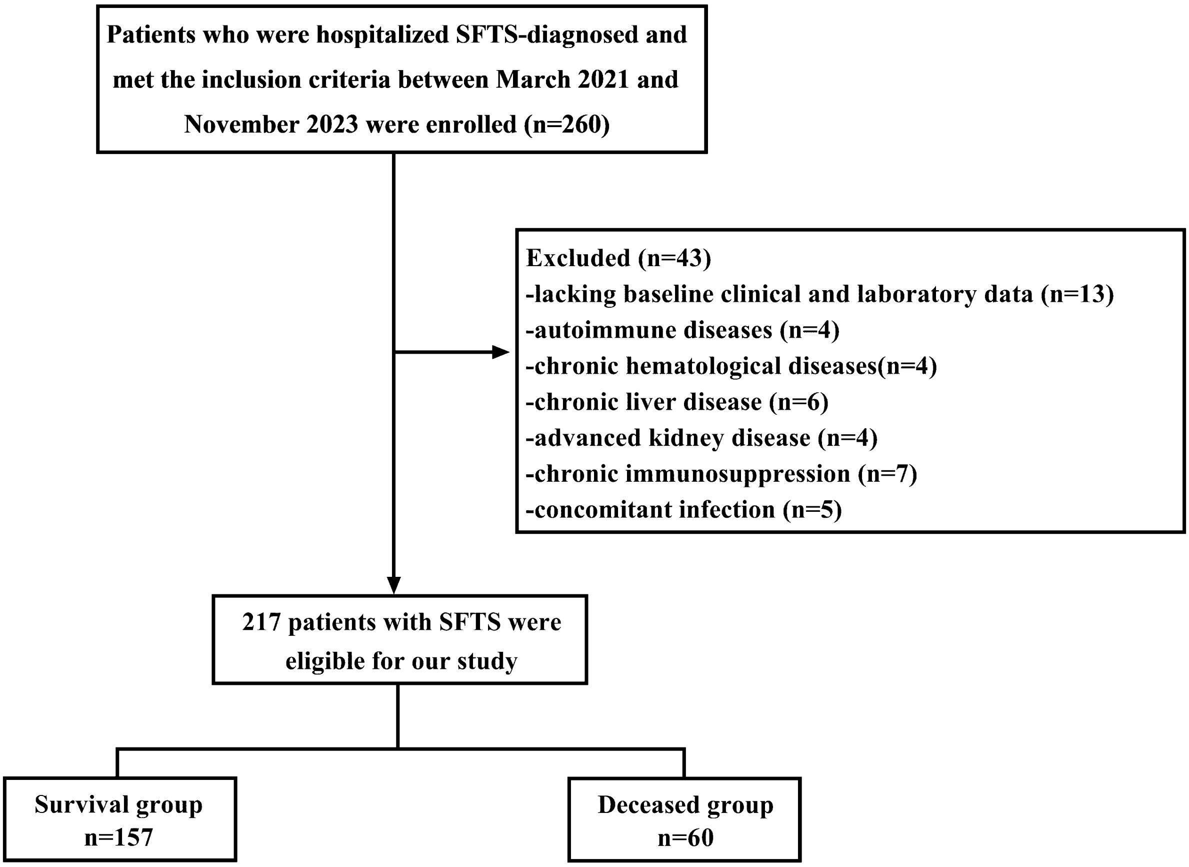

Figure S1. Flow chart of the study participants. Abbreviation: SFTS, severe fever with thrombocytopenia syndrome.

Table S1

Laboratory indexes and their abbreviation name

Full name	Abbreviation name
White blood cell	WBC
Neutrophil	NEU
Lymphocyte	LYM
Monocyte	MONO
Platelet	PLT
Serum albumin	ALB
Alkaline phosphatase	ALP
Alanine transaminase	ALT
Gamma glutamine transferase	GGT
Total bilirubin	TB
Blood urea nitrogen	BUN
Estimated glomerular filtration rate	eGFR
Creatine phosphokinase	CK
Lipase	LPS
Amylase	AMY
C-reactive protein	CRP
Procalcitonin	PCT
Blood urea nitrogen to serum albumin ratio	BAR
Activated partial thromboplastin time	APTT
Fibrinogen degradation products	FDP

Table S2

Assignment of variables in the Cox regression analysis of laboratory test indicators

**Laboratory test on admission**	**value**
WBC (x10^9/L)	*<3.5 = 1, ≥3.5 = 0*
NEU (x10^9/L)	*<1.8 = 1, ≥1.8 = 0*
LYM (x10^9/L)	*<1.1 = 1, ≥1.1 = 0*
MONO (x10^9/L)	*<0.1 = 1, ≥0.1 = 0*
PLT (x10^9/L)	*<125 = 1, ≥125 = 0*
ALB (g/dL)	*<4 = 1, ≥4 = 0*
ALP (u/L)	*>135 = 1, ≤135 = 0*
ALT (u/L)	*>40 = 1, ≤40 = 0*
GGT (u/L)	*>45 = 1, ≤45 = 0*
TB (μmol/L)	*>10 = 1, ≤10 = 0*
BUN (mg/dL)	*>20.31 = 1, ≤20.31 = 0*
eGFR (ml/ (min.1.73m^2))	*≤90 = 1, >90 = 0*
CK (u/L)	*≥200 = 1, <200 = 0*
HCO_3^- (mmol/L)	*<22 = 1, ≥22 = 0*
LPS (u/L)	*>300 = 1, ≤300 = 0*
AMY (u/L)	*>110 = 1, ≤110 = 0*
CRP (mg/dL)	*>10 = 1, ≤10 = 0*
PCT (ng/ml)	*>0.5 = 1, ≤0.5 = 0*
*BAR (mg/g)	*≥6.712 = 1, <6.712 = 0*
PT (s)	*>16 = 1, ≤16 = 0*
FDP (μg/ml)	*>5 = 1, ≤5 = 0*

* The BAR was grouped according to the optimal cut-off value.

Table S3

The difference between the validity of BAR and the other models was evaluated by the DeLong test

Parameters	Difference between areas	95% CI	*z* statistic	*p* value
BAR~				
BUN	0.051	(0.025-0.077)	3.815	<0.001
ALB	0.225	(0.145-0.306)	5.497	<0.001
CAR	0.270	(0.185-0.356)	6.213	<0.001
PAR	0.262	(0.178-0.345)	6.125	<0.001
AISI	0.252	(0.162-0.342)	5.500	<0.001

Abbreviations: BUN, blood urea nitrogen; ALB, serum albumin; BAR, BUN/ALB ratio; CAR, C - reactive protein (CRP)/ALB ratio; PAR, platelets (PLT)/ALB ratio; AISI, (neutrophils × monocytes × platelets)/lymphocytes ratio; 95% CI, confidence interval.
